# American Dental Association

**Published:** 1892-02

**Authors:** 


### AMERICAN DENTAL ASSOCIATION.

*August 6, 1891.—Third Day.—Morning Session.*

Meeting called to order at 9.45. The president in the chair.

Dr. Marshall's paper, "Pyoktanin Blue in the Treatment of Cancerous Growths," was discussed as follows:

*Dr. Waters.*—In order that there shall be no time lost, I will say a few words, as I have had a little experience in the matter. I chanced to meet with a case where there was a very offensive odor, and I asked the patient if he had been under treatment, and he said yes, for cancer. He had no one treating him then, as no one had succeeded in helping him any. I asked if they had ever relieved the odor, and he said no. I told him I thought I could remedy that. He said if I could do it, he would be very grateful. He came to my office, and, thinking that I would like to have the advice of another physician, I took some of the waste matter of the cancer between two glass slabs, and enclosed them to Dr. Day, of Boston. I got a report the next day, saying that the matter was undoubtedly from epithelial cancer. I commenced the treatment with carbolic acid, and introduced in the opening, which was big enough for my thumb to enter, a few crystals of this agent. I moved that around with a portion of cotton to bring them in contact with all surfaces. The odor at once disappeared, but there was considerable pain. I told the man to come in again in a few hours. I made another application in such a way as to catch whatever discharge there might be from it. The next day there was not so much as there had been, and no odor. The opening seemed to be larger, and the surface appeared to be a little less indurated. It did not rise up so high above the surrounding surface. I continued this treatment. In three weeks time the whole of the indurated surface had disappeared, and tissue had taken its place, and his face had nearly closed up. There was not an opening as large as a small pin-head. He came in again some time after, with his face looking worse than I had ever seen it at any time. I said to him, "What does this mean?" He said his wife wanted some wood, and he undertook to cut some hemlock wood, and a piece of it had struck him. I had to do the work of treating him all over again. Suffice it to say that the whole surface healed up under the treatment with carbolic acid, and there was no discoloration of the skin and no scar. I hold this theory in regard to cancer of any kind: that it never occurs except when the body is in a low stage of supply of certain elements.

*Dr. Truman.*—I do not propose to take up your time with the discussion of cancer, important as that may be. Under Section V., I prepared something in regard to pyoktanin, as one of the agents that was introduced during the present year; but inasmuch as Professor Marshall had brought the matter up in a paper, I concluded that I would not present the article, but make a few remarks this morning in regard to it.

Pyoktanin, as you know, was discovered or introduced by Proferror Stilling, of Strasburg, and he gave it the name, meaning pus-killer, from two Greek words. It is simply one of the coal-tar products. It is of an aniline color. Those of you who have not seen it can examine it here on the table,—the yellow and the blue. Pyoktanin may be an excellent agent for the purpose of destroying bacteria in general surgery: I have nothing to say against it there. I believe that its deep penetrating power will probably make it valuable in many directions, but in dental practice we find that this will possibly cause it to enter the tubuli, and produce coloration of the entire tooth.

I do not hold that this agent is of any great value in dental practice, as a germicide. It stains rapidly, as do all aniline dyes. It can be made in solution of one one-thousandth, one part of the agent to one thousand of water. I think the praise bestowed upon this agent by our lamented friend, Dr. Atkinson, while probably deserved in pus-pockets where there is no risk in using it, was overdrawn. I do not regard it of any more value than the ordinary derivatives from coal-tar, as naphthaline or hydronaphthol. All of the agents from coal tar-are valuable, and they are more so, in my opinion, than most of the other germicides in use.

*Dr. Fredericks.*—I should like to know whether Dr. Marshall attributes that cure solely to the employment of pyoktanin? Whether carbolic acid, for instance, would not answer the same purpose? What caused me to allude to it is this: it is well known that neoplasms sometimes are of such a growth that when the nourishment ceases they soften up, drop off, and in that manner cure themselves. In illustration of how a person can be deceived, Dr. Suchow, of the Charity Hospital of New Orleans, states that he had a case of tumor of over six months' growth. The patient was a negro. He had had trouble with it before, and had had it operated on without success. He had the tumor confined to the lower jaw, and it was of the size of a large walnut. All the roots were affected, and he concluded to extract them as the best course to pursue. This was done, but it continued to progress, and carbolic acid injections were used, and the tumor gradually decreased, and the case was cured. I think there was infiltration, although the injection of the carbolic acid, or the solution of it, say one or two per cent., ended the matter. That is the reason I asked this question. In this case the remedy employed would not prove that it was a cure for cancer; but I would like to ask Dr. Marshall in regard to it.

On motion, the discussion on this paper was passed.

Dr. Patterson then read a paper on "Fracture of the Lower Maxilla by a Gun-Shot Wound; Treatment by an Interdental Splint Bridge," of which the following is an abstract:

The patient was Sergeant Charles Campbell, of the Seventh Regiment United States regular army. In a battle with the Indians he was struck on the lower jaw by a rifle-ball, and the anterior part of the maxilla, from the second bicuspid upon the right to the second molar upon the left side, was shattered. He was given such surgical treatment as could be obtained at the time; parts of the bone and alveolar process were removed, and the wound in the lips and cheek stitched together. It was thought at one time that there would be a union of the fragments, but gradually piece after piece came away, until at one point in the region of the right cuspid, for about six inches, the bone was entirely gone, leaving the remaining parts movable. Six weeks after the accident the patient was brought to me for treatment. I found the following condition: The outside wounds healed with considerable cicatricial tissue; the left fragment of the maxilla was easily movable; an abscess was discharging at the opposite end and another abscess opening under the chin, near the symphysis; the left side of the jaw was much firmer than the right, but had healed far inside of the normal position.

I found that the bridge splint would not be of any avail, as it would result in retaining the incorrect position of the left side, and pressure brought to bear, forcing the pieces apart, would result in a greater deformity.

The first treatment was to overcome, if possible, the incorrect position of the left side. I banded the first molar remaining upon the right side, and also the first upper molar upon the same side, with lugs attached to the bands, for the reception of a screw, and firmly screwed them together. I then placed a jack-screw upon these molars on the palatal side and against the molar on the left, and forced that into its correct position. I then banded the upper and lower teeth upon this side as upon the other, and screwed them firmly together. At the end of three months the patient returned to me, and reported that he felt very comfortable, save that he was confined to soft foods. On removing the bands, the left side, after two or three days, swerved slightly inward, and then remained in almost correct position.

I then made the splint bridge, which you see in the illustration. It has been worn for six months, and the patient, whom I saw four weeks ago, says he is a new man. He can eat solid food now, and the bridge is a success. He is a young man, in good health, and the chances for growth of new bone are good.

*Dr. Brophy.*—How many teeth were removed by the accident?

*Dr. Patterson.*—All the incisors and two bicuspids on one side, and all the incisors and bicuspids on the other, and the top knocked off of one of the incisors (illustrating on the board).

*Dr. Carroll.*—This is a very interesting case, and I can illustrate it by another example. In 1864 an Irishman was shot in a battle of the late war. A minie-ball entered the left inferior maxilla, between the second bicuspid and molar, at this point (illustrating), producing a fracture here, at the angle of the ramus, between the second bicuspid and molar on the right side, and a fracture at the angle of the ramus on the right side, carrying out, at the exit of the ball, the first and second molars and second bicuspid, leaving the third molar there. A portion of the jaw was left between the second bicuspid and the ramus. The surgeons did not undertake to do anything with him, thinking the man would not live. He did, however, and came to me in February following. This had occurred in November. He was then suffering with phthisis pulmonalis, which had ensued from excessive suppuration. The front incisors on the left side had slipped past, and lay in front. Now mark: this section lay under the tongue, and he could not speak. What did I do with it? The first movement I made was to dissect up that part from under the tongue, and loosen the latter in that section. That gave me all the segments of the jaw, with the jaw loose. He took his food through a tube. I made an impression in very soft wax. I laid this down on my table, and then took an impression of the upper jaw. With both of these I made a rubber splint, and tried to get all the parts up into it, and then open it at the point of fracture. At each point there was an opening and discharge. In all fractures where we have false joints we do not necessarily have an abscess; but there was one here. From what? Necrosis of the bones lying at the ends of the fractures. I resorted to aromatic sulphuric acid, applied in full strength, and the suppuration stopped in forty-eight hours. I followed this treatment until the union of those fractures, and it caused an entire reproduction of the maxilla. The reproduction was completed within about four months. I do not remember the exact time, but I have the minutes of the case, which I shall give at some future period. Here was entire reproduction of the maxilla, which was alleged could not be done. It was a very interesting case.

*Dr. Marshall.*—I want to compliment Dr. Patterson on the way he managed this case. He made the suggestion that he was waiting very anxiously to get reproduction of bone. It occurred to me that he did not get reproduction of bone. It was a beautiful case for transplantation of bone. I have had a little experience in that line. I have been successful in one case, and not in another. I believe it might be united by transplanting bone in this manner: Laying open the tissues externally, and paring the ends of the bone, and then transplanting the bones of a child which had died at birth, or from a young rabbit half-grown, and taking pieces of the bone as thin as possible, and laying them in rows until the space was closed, and then covering it over completely, placing on the bandages. If this were done inside the mouth, it would be a failure; you would have to do it from the outside, and by putting on the bandages and keeping it perfectly aseptic, the bone would unite. In the case I mentioned not one of them failed to unite; in the other I found that I could not do so, because I did not transplant the bone, and it resulted in a failure.

*Dr. Horton.*—In connection with this case, I would say that I have a similar one, except that the trouble is of a cancerous nature, and the entire maxilla, from the second molar on the left side to the third molar on the right side, has been removed, and the patient is very anxious that something should be done. The case was that of a man who attempted to cross a trestle-work and fell and fractured his jaw. It was broken in two or three places. The surgeons having the matter in charge at the hospital sent for me to come immediately with my engine and with my drills, to perforate the jaw and put in a splint, which I had done only a few days before. I found that the tissue on the inside of the mouth had not been disturbed. I advised against perforating the jaw, and told them that if they would put on a simple plaster-of-Paris splint, that the thing could be accomplished. It was done, and proved successful.

*Dr. Patterson.*—Only one word in regard to the advisability of trying the transplantation of bone as has been mentioned. In the case referred to in the paper, I never should have advised it, from one fact alone,—that the jaw upon the right side had been too much shattered. It was in such an incomplete state that it could not have been successful. It would also have broken up the comfortable position of the patient. It was discussed with several surgeons, and it was decided that the patient should not be disturbed.

Subject passed.

*Dr. Brophy.*—The section has completed all its work, except a method of procedure that I will relate for the radical closure of cleft of the hard palate. It was not my intention when I came here to present anything to the section, as I have succeeded in securing a number of papers, and I believe the time will be quite occupied by them. I have, however, been requested to speak on this subject.

It is well known that in cases of cleft of the hard and soft palates we usually have harelip, single or double. The usual procedure is to close the fissures of the lip and let the cleft of the palate go, to be corrected later in life. This is a mistake, in my opinion. I have held this view for some time, and within the past two or three years I have attempted to make a radical operation for the closure of the hard palate. The closure of the soft palate has long been an operation well known, and, in some instances, the hard palate has also been closed by surgical procedure. The time best adapted to the closure of the latter is in early infancy, from the first to the third or fifth week after birth. We may proceed as I shall describe, and through the kindness of my friend, Dr. Jackson, we have placed upon the board a sketch, so that I may more accurately show you the operation that I am about to describe.

This operation should be made as soon as the organs of the body are performing their functions normally. Frequently, in young children suffering from this deformity, we find them extremely weak and not able to submit to an operation.

This illustrates, as you see, the palatal aspect of the superior maxillary bone. (Illustrating by means of the chart.) This represents the line of separation between the intermaxillary bone and the bone proper. We have a broad cleft of the palate here. To close that, we must resort to something more than external pressure. I am aware that Dr. Sayre, of New York, has operated by adjusting external appliances so as to exert pressure upon the sides of the face. He suggested that these operations might be made for the closure of the palate, since in the early infant the bones are so soft that they may be easily bent in narrow clefts, so that the edges may be approximated. But in this case it is absolutely impossible by external pressure to get the parts in proximity. The method that I have suggested is to first close the soft palate in the usual way, by paring well the borders and closing it with wire. Having freshened the edges of the hard palate, we then lift the lips and the cheeks, and insert a drill, carrying it through the substance of the maxillary bone, so that it will pass through above the palatal blade. Then beginning with the drill upon the opposite side, carry it through so it will have a small opening from side to side, anterior to the malar process. Then insert the drill back of the malar process, carrying it through in the same manner, so that it will pass above the palatal blade or bone. Having done this, we introduce a strong wire, and carry it through the bone-substance. Then we make use of a lead button; this is perforated, so we may lay it upon the sides of the bone, as shown here,—this representing the buccal surface of the jaw,—and introduce the wire through one of these openings. Now we set those wires up by twisting them together. This is done because it is impossible to exert sufficient pressure to get the parts in proximity. Then with a knife the cheek is lifted and the bone is divided, so as to separate it absolutely from the tissues above. Do this upon either side. Having separated the maxillary bone from the tissues above, we have then no trouble in twisting our wires and setting them up and bringing them in close proximity. By so doing, we will get a union of the bone on either side.

The question may be raised, What will be the effect upon the germs of the undeveloped teeth? That I do not know. I have one case far enough advanced, so that we will soon see what the results will be. Even if the germs of several teeth are destroyed, what is that in connection with the fact of being relieved of such a great deformity. Everything, gentlemen, is in favor of this early operation. The child, in the first place, after the parts are closed, is better nourished. The parts are more easily manipulated. The operation can be made with less shock to the patient, inasmuch as the nervous system of the child is not so highly developed as later. Another thing is that when the child comes to an age when he may articulate, this is correctly performed. He has not the difficulty to overcome, as children sometimes do, and he lives and grows without being embarrassed through life with this great deformity. I would have preferred to write something on this, and to present the matter more concisely, but the section has already occupied a great deal of time, and I will close with what I have said.

*Dr. Truman.*—What was the age of the patient operated upon?

*Dr. Brophy.*—Three weeks is the age I prefer. I think that if there are any questions that gentlemen would like to ask, that they should be permitted.

*Dr. Marshall.*—I would like to ask why this operation is any better than the one introduced by Ferguson. It certainly is a more serious operation. Dr. Brophy made the statement that the nervous system of a child of that age was not so highly organized as later in life. The fact, I believe, is, and it is almost universally conceded to be, that shock to a little child is much greater than to an older individual, by the same operation. Serious operations are never performed on little children. It is usually put off until after dentition. It seems to me that, in a case of that kind, it would be better to wait until after dentition is completed, and then make the operation in this way (illustrating):

Simply divide the mucous membrane about midway between the edge of the cleft and the inner aspect of the teeth, on both sides, and then, paring the edges, lift the periosteum and suture them together. In a number of cases you will have the formation of bone. That is less serious to a child than the operation Dr. Brophy has indicated. I should think to perform such an operation on a little child would be almost certain death. I am of the opinion that, if he keep a record of his cases, it will almost always prove fatal.

Another way would be to drill the bone and split it, which would be still less shock to the child than would be the practice proposed. I cannot see how that is an improvement upon the old operation.

*Dr. Patterson.*—I thought it well settled that staphylorrhaphy in the dental profession had been for many years discarded, for the reason that the perfection of articulation is never accomplished by that operation. It is impossible to close the opening whenever there is insufficiency of soft tissues present, and perfect articulation cannot be had.

*Dr. Volck.*—I have operated on a great number of patients, on the hard and the soft palate. I have had many cases, and I think that operating on the hard palate on young children is a very serious thing. I have seen two of them die from the effects, and I attribute it to the nervous shock. The operation on the soft palate, as our friend tells us, is in most cases successful. The tissue accommodates itself as the child grows, so that the articulation is almost perfect.

I do not give this as a speculation. I give it as a matter of positive experience. The operation on the hard palate, I was on the point of remarking, is very ingenious, but I do not believe a child could stand it. The other which I have performed was done by separating part of the hard palate from the rest, drawing it together.

*Dr. Marshall.*—With regard to closure of the soft palate, a statement has been made that it is never successful, as far as speech is concerned. That depends on the operation. I say positively that if properly performed, with a sufficient amount of soft tissue, it is generally successful. I have seen a number of cases that have been recommended to wear an artificial piece, which I thought could be better treated by the knife. I will give you one illustration, that of an Irishwoman, forty-nine years old, who came to this country four years ago and went to Boston, and was in the hands of one of the best men we have there. He made her an artificial plate, and after wearing it three or four months she said she could see no change in her speech. She finally came to Chicago, and was sent to me. I tried to understand what that woman said, but she had to write it. I told her to take out the piece, and she did, and I could not distinguish the difference. I said to her, "I think you can be helped by an operation. I do not say this because I desire to perform it, but I think it will really help you." She said, "Anything you recommend I will submit to." I said, "I can put your mouth in as good condition as it is in now, discarding your plate." She consented to it, and if I had her here to-day, there is not a person but could understand her perfectly. She does not talk with absolute clearness, but sufficiently so to be satisfactory.

I operated in this way: I divided the mucous membrane and lifted it up, freshening the edges of the soft palate, and then, when that was completed, I took my knife and cut right through the soft palate, so as to loosen it. That separated and did not unite. She cannot swallow liquids without having them enter the nasal passage.

*Dr. Volck.*—My way of practice was this: The flap hangs about this way (illustrating); to bring it entirely together is almost impossible; but by separating, from the inside, the velum slightly from the bone, and then slitting it not entirely through, you can get the flaps almost through. They generally hang down, and to bring them together is difficult. Even if brought together, the hard palate beyond these ends will draw back when forced together. The operation is a failure. But if the scalpel is passed under this, and it is then separated from the bone and split, it can be drawn together with great ease, and will heal perfectly. In such an operation on a grown person the difficulty is still greater. I must confess that I have had only partial success in operating on the palates of those who have reached maturity.

*Dr. Brophy.*—I am highly gratified with this discussion, because it has brought out some features of the operation on the hard and soft palates that I could not touch upon, and which I will be pleased to answer. Dr. Marshall's remarks, as to the shock caused upon young children, I do not think well established by our knowledge of physiology. The works on physiology are the best authorities to show us that the young child is not so susceptible to nervous shock as one further developed in life. I refer you to standard works in verification of this statement.

As to the next question about the shock produced in this operation, I will answer that our best knowledge is that based upon clinical experience. I have had seventeen cases, in three of which this operation was used, and in not a single instance has there been inflammation. In verification of this, I can exhibit certain patients upon whom I have operated in Chicago. The statement made by Dr. Patterson is one I should possibly have made myself, but I did not enter into all details. I could not for want of time. The question is as to the lack of soft tissue. It is a fact that a child having cleft of the palate when it is born, has, in nearly all instances, as much of a soft palate as any child that is perfect, only the surfaces are not united. If the child be permitted to go on, the soft tissues are all put to use. It is not necessary for me to state that all the parts of the body must be used to be nourished. It is well known that the right arm of the blacksmith is stronger than his left arm, because he uses it more. It is a fact that if the soft palate is closed in a small child it will not develop.

It seems scarcely necessary to speak of this, and the difficulty is that the tissues are not properly brought together and are not well secured.

The usual operation is to carry it around so (illustrating); the tissues must be brought together in this way, so that they will not be allowed to slip back in speaking or crying. There is where the mistake lies,—the carrying of the loop around, and these two flaps not being allowed to unite. If the tissues are not united, if the soft palate is permitted to go without union, there is an atrophy of the parts for want of use. Hence, later in life, as clinical experience shows, the operations have been a failure, because the tissues have atrophied, and we have not enough to bring together and close the posterior nares in speech. We must have sufficient tissue to close these to produce articulation. Later in life it cannot be done; hence the failure.

I am satisfied many a patient is wearing an artificial palate who, had he been operated on early in life, would never have been obliged to submit to such a thing.

There is another feature: some gentleman has said we should wait until the teeth have developed. Do not take those statements down and practise them until you have given this careful thought. Let the teeth be developed and the articulation established, and you cannot make an operation and bring the maxillary bones together without disturbing the articulation. When made early in infancy, it does not effect it to any great extent. If the arch should be contracted, it may be easily expanded. This operation I have performed a great many times by loosening the bone and bringing it over. I regard it as very inferior, however. Later in life we have dense tissues, highly organized, with blood-vessels and nerves. A shock is produced that would not be if the soft parts were brought together when almost in a jelly-like form. This is all I can say about it.

